# Supplementary material for: Impacts of Nanobubbles in Pore Water on Heavy Metal Pollutant Release from Contaminated Soil Columns
Source: Nanomaterials (Basel). 2023 May 18;13(10):1671. doi: 10.3390/nano13101671 (PMC10222895; doi:10.3390/nano13101671)
Supplement: Supplementary file 1 [file nanomaterials-13-01671-s001.zip › nanomaterials-2380555-supplementary.pdf]

Supporting Information

For

Impacts of Nanobubbles in Pore Water on Heavy Metal Pollutant Release from Contaminated Soil Columns

Yihan Zhang [1], Zimu Song [1], Kosuke Sugita [2], Shan Xue [1] and Wen Zhang [1,*]

[1] Department of Civil and Environmental Engineering, New Jersey Institute of Technology, Newark, NJ 07102, USA; yz27@njit.edu (Y.Z.); zs6167575@gmail.com (Z.S.); sx59@njit.edu (S.X.)

[2] Department of Mathematical Sciences, New Jersey Institute of Technology, Newark, NJ 07102, USA; ks823@njit.edu

* Correspondence: wen.zhang@njit.edu

Contents

Supporting Information........1

S1. Chemicals used in this study.........2

S2. Heavy metal analysis using ICP-MS........2

S4. Measurements of nanobubble water surface tension using pendant drop method........4

S4. Measurements of nanobubble water surface tension using pendant drop method........5

S5. The impact of CO_2 partial pressure on the ORP of the soil elute........6

S6. Utilization of CDDE and CDE models and relevant reults........7

References........16

S1. Chemicals used in this study.

Table S1. The basic properties of the Miracle Gro Garden Soil

**Soil texture**	**Sandy Loam**		
**Soil index**	Unit	Value	Level
**pH**	Dimensionless	5.76	Moderately Acidic
**Phosphorus**	Pounds per acre	121.5	optimum
**Potassium**	Pounds per acre	138	Below optimum
**Magnesium**	Pounds per acre	304.5	Above optimum
**Calcium**	Pounds per acre	2738.5	Above Optimum
**Zinc**	ppm	3.97	Adequate
**Copper**	ppm	3.69	Adequate
**Manganese**	ppm	109.1	High
**Boron**	ppm	1.02	Adequate
**Iron**	ppm	219.95	High
**Electrical conductivity**	mmho·cm^{-1}	0.4	Satisfactory
**Organic Matter by loss on ignition**	%	3.0	High for sandy Loam
**Gravel**	%	30.2	Larger than 2 mm
**Sand**	%	78	Sandy Loam
**Silt**	%	13	Sandy Loam
**Clay**	%	10	Sandy Loam

S2. Heavy metal analysis using ICP-MS

The supernatant was filtered through the 0.45 μm membrane to remove any soil or large debris. The dissolved heavy metal ions [1] was subject to Inductively Coupled Plasma Mass Spectrometry (ICP-MS) analysis following EPA standard method 6010 with the corresponding MDL ranges varying between 0.1 and 0.5 ppm.[2] Instrument tuning was performed prior to analysis by using a EPA Method 200.8 Calibration Standard solution (ICP-200.8-1-100, hps, USA) according to the manufacturer's recommendation.

For liquid samples, 10 mL of filtered supernatant was acidified by adding 1.5 ml of 60-70% TraceMetal grade nitric acid (A509-P212, Fisher Chemical, Canada) to keep the pH under 2. Then all the samples were incubated in a hot block (SC151-240 Digestion System, Environmental Express, USA) for digestion and evaporation until the volume of each sample has been reduced to the original 10 mL. In this work, the digestion lasted for 4 hours, and the temperature of the samples were maintained at 85-95°C.

To determine the Pb concentration in soil, soil acid digestion was conducted following the modified EPA standard method 3051A.[3] Before digestion, every soil sample was grinded and sieved with a 2-mm mesh. Briefly, 0.1 g of sample and 10 mL of 60-70% TraceMetal grade nitric acid were added into a 250 mL digestion vessel. All glass vials were heated to 145°C for 80 min in a microwave digestion system (MiniWave, SCP SCIENCE, USA). After the sample was cooled, DI water was used to wash out the solution until the volume reached 50 mL. Particulates in the digestate were removed by centrifugation, or by allowing the sample settle overnight.[4]

Positive control: the Pb reference solution (the Pb salt solution used to spike the soil) with a known concentration (226 mg·L^{-1}) was subject to ICP-MS analysis. Quality control samples including solvent blank, spiked blank, spiked matrix duplicates, and sample duplicates were routinely determined together with the samples.[5]

S3. The nanobubble water generation method

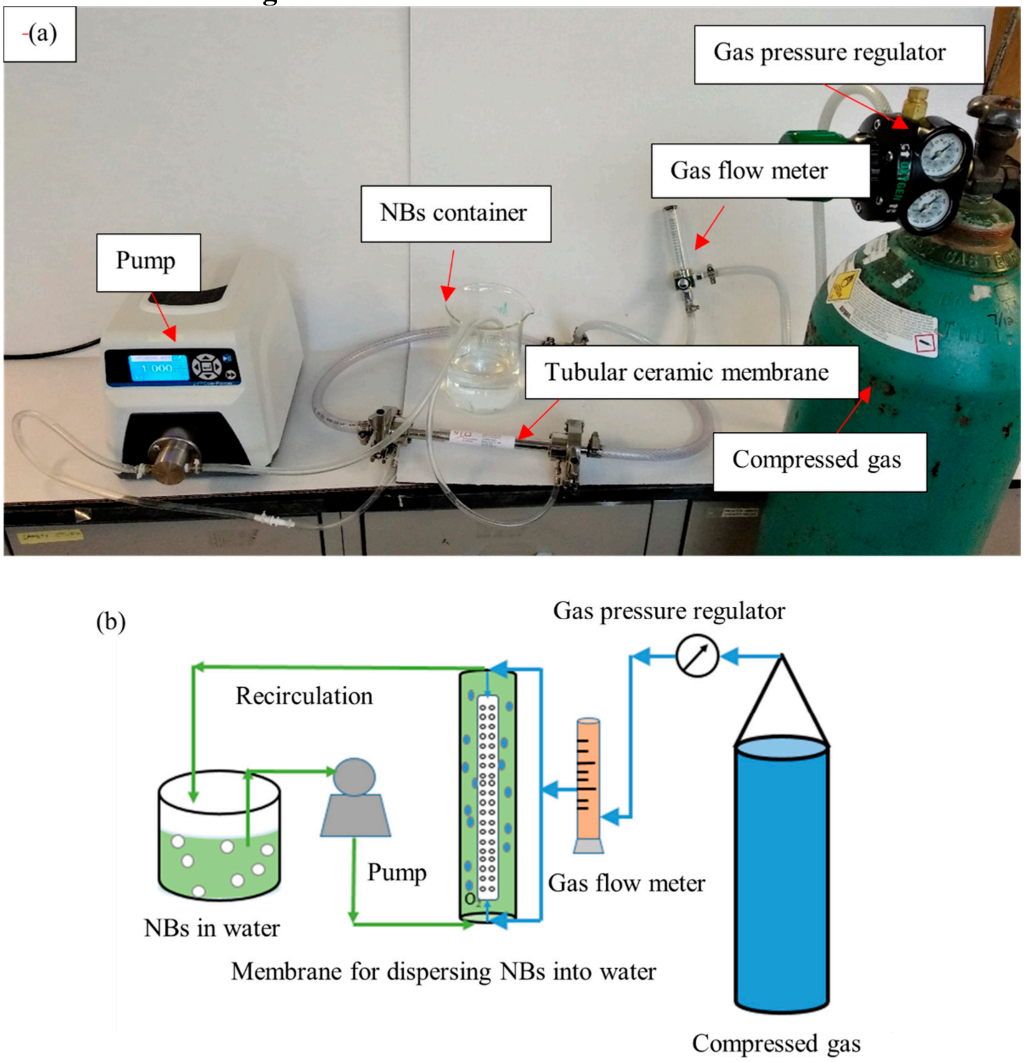

Figure S1. (a) Photo of the nanobubble generator system; (b) Schematic of the generation process.

S3. Measurements of nanobubble water surface tension using pendant drop method

The experimental apparatus required for pendant drop tensiometry is shown in **Figure. S2a**,[6] where a needle with an inner diameter of 0.5 mm, a camera, and a light source were assembled. The light source must be diffused to ensure that no optical aberrations occur at the drop periphery, and spurious reflections from the drop interface arising from other sources (e.g., overhead lighting) must be avoided. Similarly, the drop image captured at the digital camera sensor must be undistorted by lensing effects, and analysis is greatly simplified by a homogeneous image background. A typical image in **Figure S2b** was used to determine the geometry information for calculation. Finally, the requirement of droplet axisymmetry is central to obtaining precise measurements of interfacial tension, and thus the needle must be absolutely vertical.[7]

A drop of the sample liquid formed at the tip of the needle and imaged at a frequency of one drop every 2 s. For each nanobubble water sample, about 15 images were taken.[8] Images were analyzed using ImageJ to obtain the drop radius (R_o). The surface tension (γ, $N \cdot m^{-1}$) was calculated using Eq. (S1):

$$\gamma = \frac{\Delta \rho g R_O^2}{B_O} \quad \text{(S1)}$$

where $\Delta\rho$ is the density difference between the liquid drop (991.5-997 $kg \cdot m^{-3}$) and the ambient air (1.293 $kg \cdot m^{-3}$), g is gravitational acceleration (9.81 $N \cdot kg^{-1}$); B_O is the abbreviation for Bond number, which measures the relative importance of gravitational forces to interfacial forces. To determine the Bond number, DI water was first tested to obtain the R_o, and the B_O was calculated and determined to be 0.02 using the Eq. (S1) with the DI water surface tension of 72 $mN \cdot m^{-1}$ at room temperature. Since the same needle was used with a diameter of 0.5 mm, the same B_O value could be used in the surface tension calculation for different NB waters.

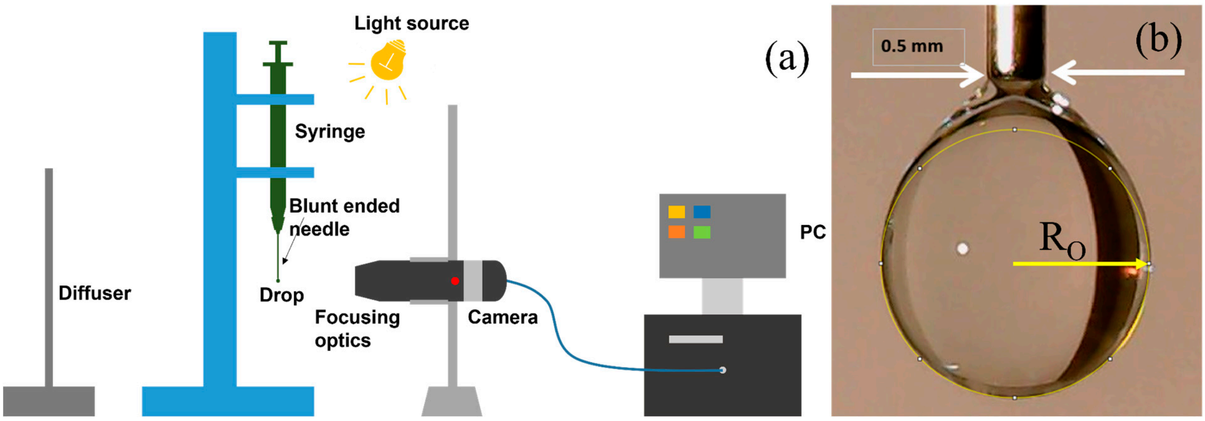

Figure S2. (a) experimental apparatus of the pendant drop method; (b) photo of the drop captured at the tip of the needle.

S4. Calculation of upflow water flow velocity

The upflow water flow velocity affects the soil column structures (e.g., fluidization) and according to Eq. (S2), the minimum flow velocity that can overcome the weight of the media (i.e., soil) and raise it into the water can be calculated.[9]

$$\upsilon_{mf} = \frac{\mu}{\rho d_{90}}(33.7^2 + 0.0408Ga)^{0.5} - \frac{33.7\mu}{\rho d_{90}} \quad \text{(S2)}$$

where υ_{mf} is the minimum fluidization velocity (m·s^{-1}); g is the acceleration of gravity (m·s^{-2}); μ is the dynamic viscosity of the fluid (Pa·s); ρ is the mass density of the fluid or water (kg·m^{-3}), d_{90} describes the diameter of the soil grain, [10] where 90 percent of the distribution has a smaller particle size and 10 percent has a larger particle size. Ga is the Galileo number that is calculated by Eq. (S3):[9]

$$Ga = d_{90}^3 \frac{\rho(\rho_s - \rho)g}{\mu^2} \quad \text{(S3)}$$

where ρ_s is the density of the soil particles (1130 kg·m^{-3}).

Figure S3a shows the grain size distribution for the soil we used in the column. The d_{90} of the used soil is 1.62±0.01 mm; The effective size E, particle size that correspond to the 10% of cumulative mass that passes through the sieves, is 0.1 mm; and the uniformity coefficient $U = \frac{P_{60}}{P_{10}} = 3.72$. Considering the large size variations, we calculated the v_{mf} values for a reasonable range of grain sizes covering 0.3-1.62 mm and plot the results in **Figure S3b**. The v_{mf} value for the grain size of 1.62 mm is 0.0019 m·s^{-1}, which corresponds to a flow rate of 81.67 mL·min^{-1}, which is way higher than the constant flow rate (5 mL·min^{-1}) decided experimentally from above.

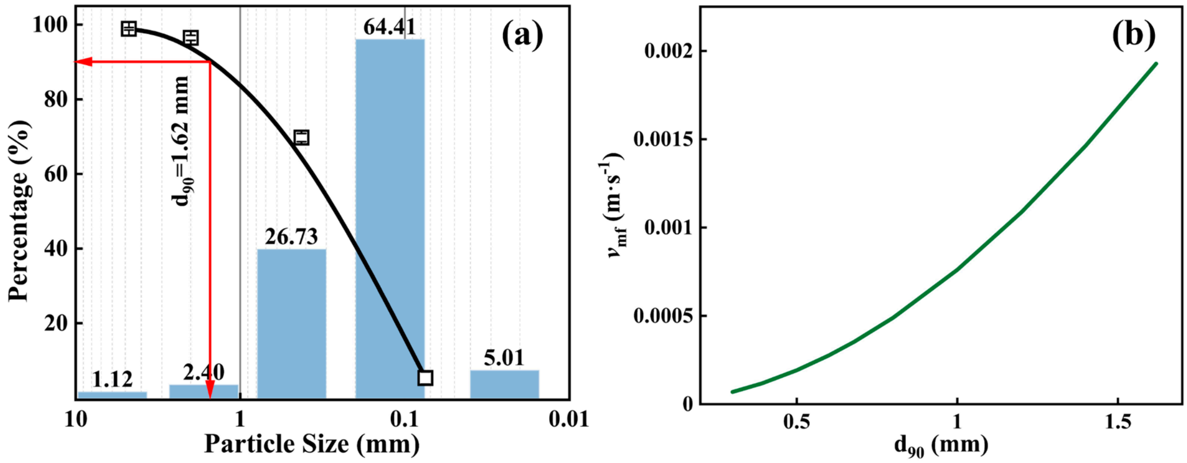

Figure S3. (a) Particle size distribution of the soil grains determined by sieving. (b) The minimum fluidization velocity as a function of grain size or d_{90}.

S5. The impact of CO_2 partial pressure on the ORP of the soil elute

The change of ORP in the soil elute under the CO_2 NB water could be partially explained by many possible half reactions as listed below, which may happen to CO_2 depending on specific chemical environment.

$CO_2(g)+2H^{+}+2e^{-} \rightarrow HCOOH(l)$; E_0 = -0.61 V

$CO_2(g)+H_2O(l)+2e^{-} \rightarrow HCOO^{-}(aq)+OH^{-}$; E_0 = -0.43 V

$CO_2(g)+2H^{+}+2e^{-} \rightarrow CO(g)+H_2O(l)$; E_0 = -0.53 V

$CO_2(g)+H_2O(l)+2e^{-} \rightarrow CO(g)+2OH^{-}$; E_0 = -0.52 V

$CO_2(g)+4H^{+}+2e^{-} \rightarrow HCHO(l)+H_2O(l)$; E_0 = -0.48 V

$CO_2(g)+3H_2O(l)+4e^{-} \rightarrow HCHO(l)+4OH^{-}$; E_0 = -0.89 V

$CO_2(g)+6H^{+}+6e^{-} \rightarrow CH_3OH(l)+H_2O(l)$; E_0 = -0.38 V

$CO_2(g)+5H_2O(l)+6e^{-} \rightarrow CH_3OH(l)+6OH^{-}$; E_0 = -0.81 V

$CO_2(g)+8H^{+}+8e^{-} \rightarrow CH_4(g)+2H_2O(l)$; E_0 = -0.24 V

$CO_2(g)+6H_2O(l)+8e^{-} \rightarrow CH_4(g)+8OH^{-}$; E_0 = -0.25 V

$2CO_2(g)+12H^{+}+12e^{-} \rightarrow C_2H_4(g)+4H_2O(l)$; E_0 = 0.06 V

$2CO_2(g)+8H_2O(l)+12e^{-} \rightarrow C_2H_4(g)+12OH^{-}$; E_0 = -0.34 V

$2CO_2(g)+12H^{+}+12e^{-} \rightarrow CH_3CH_2OH(l)+3H_2O(l)$; E_0 = 0.08 V

$2CO_2(g)+9H_2O(l)+12e^{-} \rightarrow CH_3CH_2OH(l)+12OH^{-}$; E_0 = -0.33 V

For example, the first redox half reaction indicates the oxidation of CO_2 to HCOOH. The E_H-pH relation for this reaction is written as:

$$E_H = E^0 + (\frac{0.059}{4})\{\log(P_{CO_2}) - 4pH\} \quad \text{(S4)}$$

where E^0 = -0.61 V, P_{CO_2} = 0.00038 atm for ambient CO_2, and pH = 5.5, So E_H = -0.98 V. All the other related half reactions generally lead to the the same dependence of ORP on the CO_2 vapor pressure.

S6. Utilization of CDDE and CDE models and relevant reults

To employ the CDDE or CDE model equations to calculate the time-resolved leached concentration (C_t), the following model parameters must be determined or estimated.

(1) The retardation factor (R_f) for aqueous-phase transport of solute undergoing convection, diffusion and partitioning to soil is given by:[11, 12]

$$R_f = 1 + H\theta_a/\theta_w \quad \text{(S5)}$$

where H is the air-water partition (Henry's) coefficient for lead (0.0015[13, 14]), θ_a is the ratio of the volume of air to the total volume (0.067), and θ_w is the ratio of the volume of water to the total volume (0.41). θ_a and θ_w were determined experimentally by measuring the amount of DI water or NB water it takes to fill all the tiny pores in the soil layer.

(2) The hydrodynamic dispersion coefficient for electrolytes in absence of electric field can be estimated as follows:[15]

$$D = \frac{RT}{F^2}\left[\frac{1/n^{+}+1/n^{-}}{1/\lambda_{+}^{\circ}+1/\lambda_{-}^{\circ}}\right] \quad \text{(S6)}$$

where R is the universal gas constant, 8.314 J·(mol·K)$^{-1}$, T is the absolute temperature K (273 + °C), F is Faraday's constant, 96,500 C·eq^{-1}, n^{+} is cation valence (2 for lead), n^{-} is anion valence (1 for Chloride), λ_{+}° is the limiting positive ionic conductance (69.5 for lead), cm^2·S·eq^{-1}, λ_{-}° is the limiting negative ionic conductance (76.4 for Chloride), cm^2·S·eq^{-1}. According to Eq. (S6), the hydrodynamic dispersion coefficient for $PbCl_2$ is estimated to be 1.45×10^{-5} cm^2·min^{-1}.

(3) The deposition rate coefficient (K_d) is calculated by:

$$K_d = -\frac{v}{L}\ln(MR) \quad \text{(S7)}$$

Where v is the average water velocity of the pore water flow, L is the soil column length (5 cm), and MR (%) is the effluent mass recovery rate, which is the mass percentage of Pb washed out in the 2-h experiments over the total Pb mass in the soil.

To solve the CDDE or CDE equations, we applied the following strategies. Since the travel distance $x = vt$ and $\frac{dx}{dt} = v$, the partial derivatives can be rewritten as derivatives with respect to only t eliminating x:

$$\frac{\partial C}{\partial t} = \frac{dC}{dt} \quad \text{(S8)}$$

Also, we know $\frac{\partial C}{\partial x} = \frac{dC}{dx} = \frac{dC}{dt}\frac{dt}{dx} = \frac{1}{v}\frac{dC}{dt}$ (S9)

Thus,

$$\frac{\partial^2 C}{\partial x^2} = \frac{d^2 C}{dx^2} = \frac{1}{v^2}\frac{d^2 C}{dt^2}$$

(S10)

The CDE can be rewritten as:

$$Rf\frac{dC}{dt} = D\,\frac{1}{v^2}\frac{d^2C}{dt^2} - \frac{dC}{dt} - K_d\,C \text{ or } \frac{D}{v^2}\frac{d^2C}{dt^2} - (Rf+1)\frac{dC}{dt} - K_d\,C = 0 \quad \text{(S11)}$$

This Eq. (11) is a second order linear ODE with a general solution shown below:

$$C(t) = A^{+}e^{\gamma^{+}t} + A^{-}e^{\gamma^{-}t} \quad \text{(S12)}$$

where $A^{\pm}$ are constants, and

$$\gamma^{\pm} = \frac{v^2}{2D}\left(Rf + 1 \pm \sqrt{\delta}\right) \quad \text{(S13)}$$

where

$$\delta = (Rf + 1)^2 + \frac{4D}{v^2} K_d \tag{S14}$$

Noting that $\gamma^+>0$ and $\gamma^-<0$, and that our model has to decay as t increases to capture the behavior of transport of solutes, A^+ must be zero. Otherwise, no analytical solution is found.

Let C_0 be the initial value of C(t) at t = 0. So $C_0 = A^-$. Therefore, we obtain the exponentially decaying solution:

$$C(t) = C_0\, e^{\gamma^- t} \tag{S15}$$

Similarly, we can eliminate the variable x in the CDDE, leading to:

$$Rf \frac{d^\alpha C}{dt^\alpha} = \frac{D}{v^2} \frac{d^2 C}{dt^2} - \frac{dC}{dt} - K_d\, C \tag{S16}$$

The solution can be computed numerically. We apply the same L1 method for the Caputo fractional derivative to this equation.

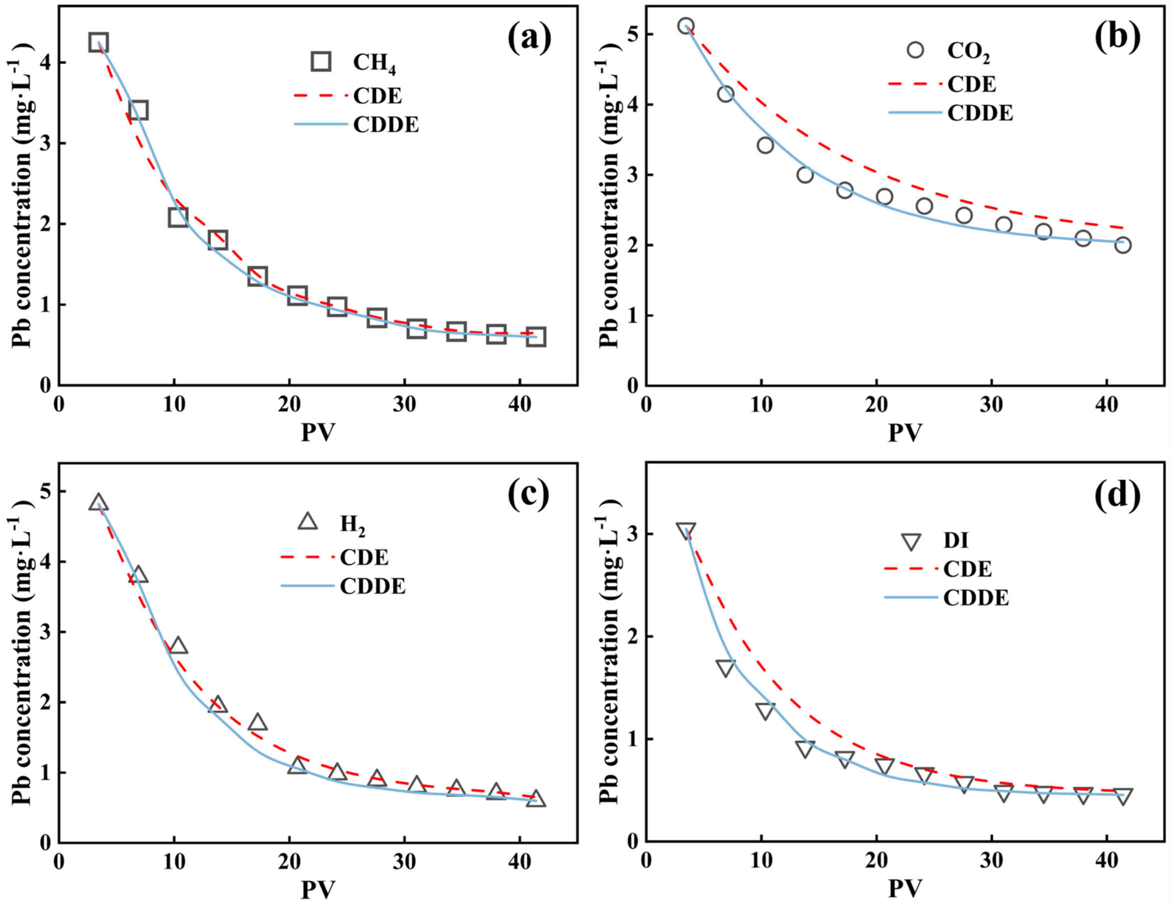

Figure S4. Breakthrough curves of Pb under a water flow containing: (a) CH_4 NBs; (b) CO_2 NBs; (c) H_2 NBs; (d) DI water. When varying K_d, the parameter α=1. The fitting parameters for the CDDE model fitting: α=0.97 and K_d= 0.07974 min^{-1} for CH_4; α=0.97 and K_d= 0.0462 min^{-1} for CO_2; α=0.97 and K_d= 0.07602 min^{-1} for H_2; and α=0.97 and Kd= 0.09252 min^{-1} for DI water. The fitting parameters for the CDE model fitting (α=1): K_d= 0.07974 min^{-1} for CH_4; K_d= 0.0462 min^{-1} for CO_2; K_d= 0.07602 min^{-1} for H_2; and K_d= 0.09252 min^{-1} for DI water.

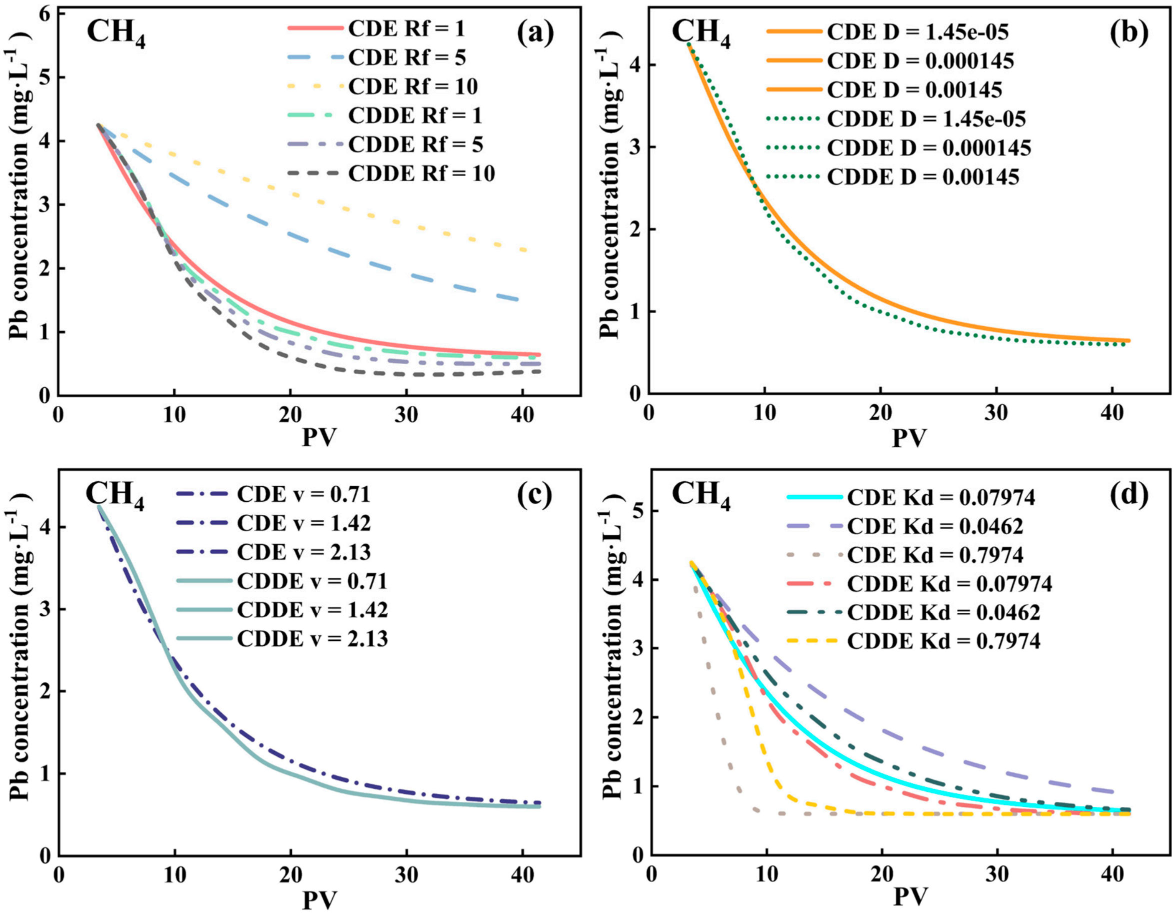

Figure S5. Model prediction of the leached Pb concentrations under CH_4 NB water wash using the CDDE model. (a) The results when varying R_f; (b) The results when varying D; (c) The results when varying v; and (d) The results when varying K_d. When changing one specific parameter, other parameters were constant unless specified (i.e., α=0.97, D= 1.45×10^{-5} $cm^2\cdot min^{-1}$, v= 0.71 $mL\cdot min^{-1}\cdot cm^{-2}$, and K_d= 0.07974 min^{-1}), which also applies to the following data graphs in **Figure S6-S8**.

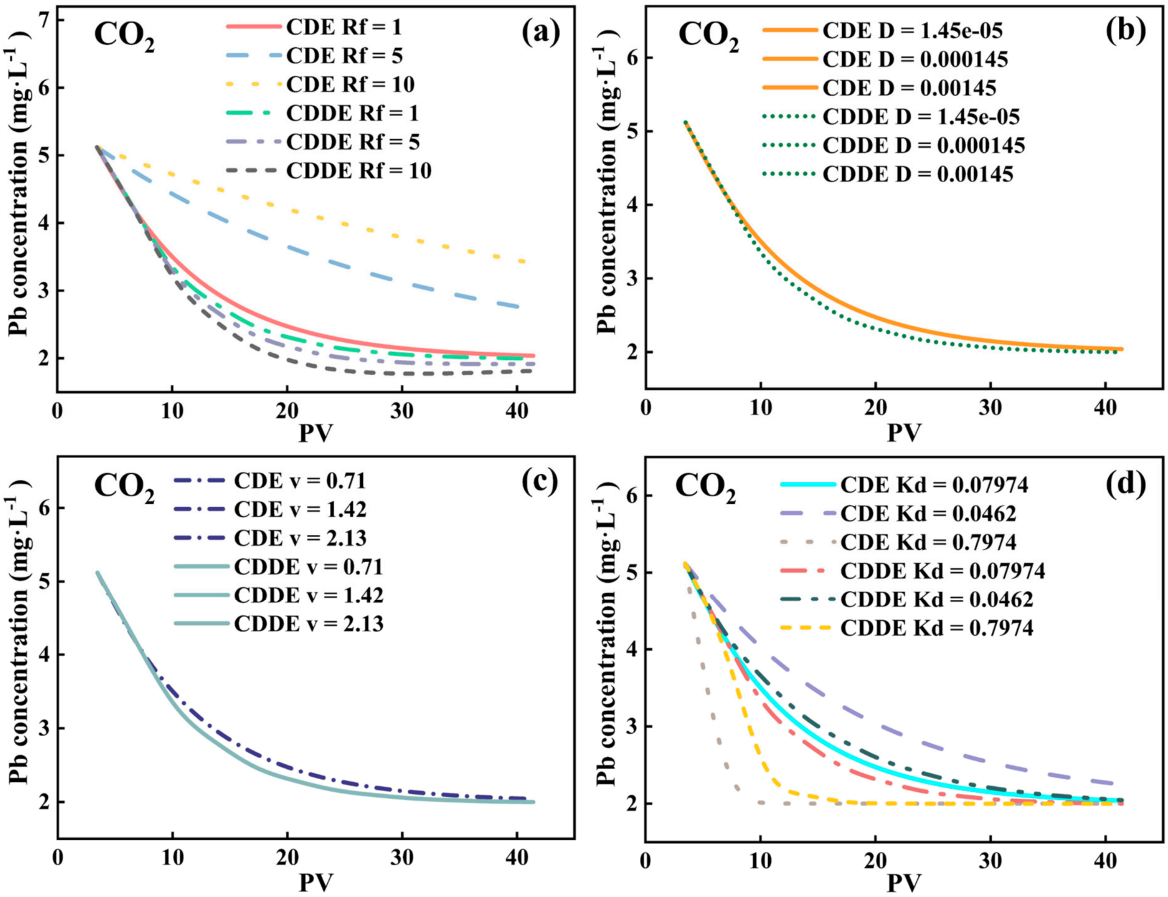

Figure S6. Model prediction of the leached Pb concentrations under CO_2 NB water wash using the CDDE model. (a) The results when varying R_f; (b) The results when varying D; (c) The results when varying v; and (d) The results when varying K_d. When changing one specific parameter, other parameters were constant unless specified (i.e., α=0.97, D= 1.45×10^{-5} $cm^2\cdot min^{-1}$, v= 0.71 $mL\cdot min^{-1}\cdot cm^{-2}$, and K_d= 0.07974 min^{-1}).

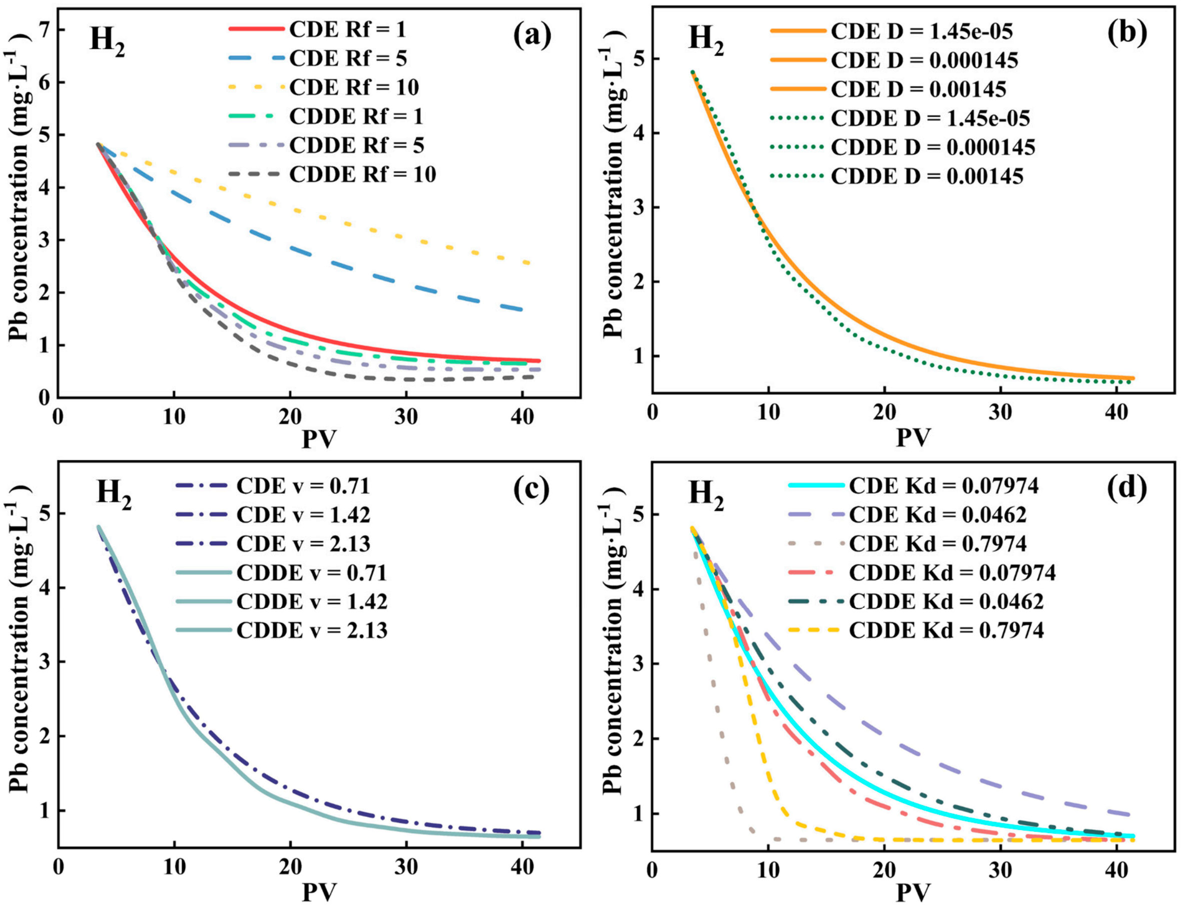

Figure S7. Model prediction of the leached Pb concentrations under H_2 NB water wash using the CDDE model. (a) The results when varying R_f; (b) The results when varying D; (c) The results when varying v; and (d) The results when varying K_d. When changing one specific parameter, other parameters were constant unless specified (i.e., α=0.97, D= 1.45×10^{-5} $cm^2\cdot min^{-1}$, v= 0.71 $mL\cdot min^{-1}\cdot cm^{-2}$, and K_d= 0.07974 min^{-1}).

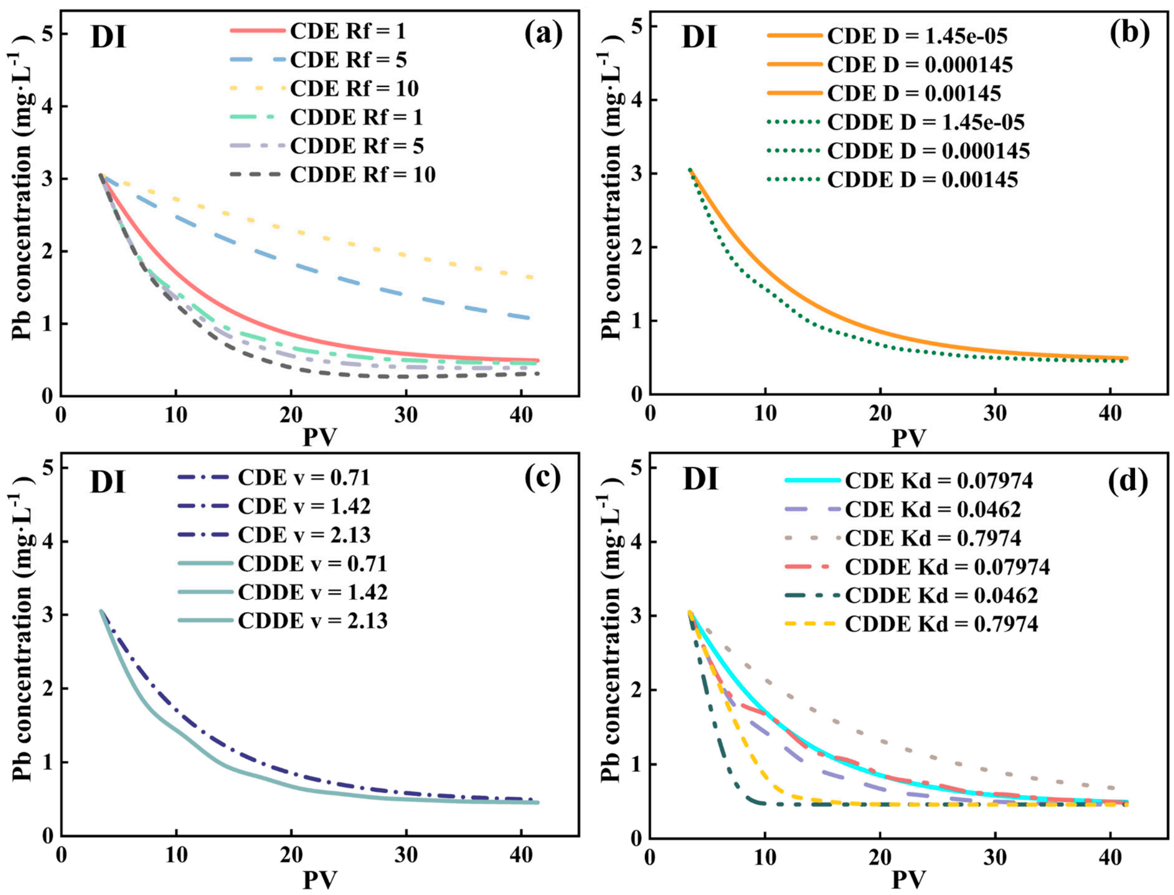

Figure S8. Model prediction of the leached Pb concentrations under DI water wash using the CDDE model. (a) The results when varying R_f; (b) The results when varying D; (c) The results when varying v; and (d) The results when varying K_d. When changing one specific parameter, other parameters were constant unless specified (i.e., α=0.97, D= 1.45×10^{-5} $cm^2\cdot min^{-1}$, v= 0.71 $mL\cdot min^{-1}\cdot cm^{-2}$, and K_d= 0.07974 min^{-1}).

Figure S9 is the simulation results using CDDE model to evaluate the impact of time fractional derivative (α). It shows that when α increases from 0.97 to 1.0, the leaching velocity increases. When using a time fractional derivative, which takes into account the non-integer order of diffusion that occurs when particles are moving through a porous medium. When α is increased, it implies that the diffusion process is becoming more anomalous and therefore the leaching process is more sensitive to changes in the concentration of the solute in the liquid phase. In other words, the leaching process becomes more efficient and the rate of extraction of the solute form the solid material increases.

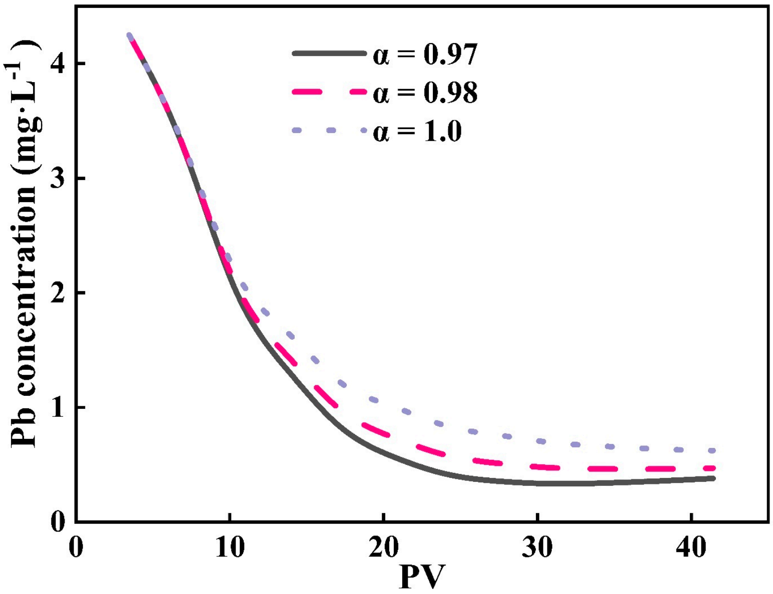

Figure S9. Model prediction of the leached Pb concentrations under CH_4 NBs water wash using the CDDE model. (D= 1.45×10^{-5} $cm^2\cdot min^{-1}$, v= 0.71 $mL\cdot min^{-1}\cdot cm^{-2}$, and K_d= 0.07974 min^{-1}).

Table S2. The parameters of the CDE and CDDE model for fitting the experimentally measured concentrations of Pb or other metals.

Parameters used in the context	Descriptions	Values or determination methods	Units
C_t	Aqueous concentration of heavy metals leached from the soil	Measured experimentally or calculated by the CDDE or CDE models	$mg \cdot L^{-1}$
R_f	The retardation factor for aqueous-phase transport of solute undergoing convection, diffusion and partitioning to soil [11]	$R_f = 1 + H\theta_a/\theta_w = 1 + 0.0015 \times 0.07/0.41 \approx 1.00$	—
α	Time fractional derivative order (between 0 and 1)	α is obtained through the fitting processes in the CDDE model or equal to 1 when using the CDE model.	—
H	The air-water partition (Henry's) coefficient	0.0015 for Pb[1541, 42]	—
θ_a	The ratio of the volume of air to the total volume, volumetric water content	$\theta_a = \frac{V_a}{V_S+V_w+V_a}$ =0.07	—
θ_w	The ratio of the volume of water to the total volume, volumetric water content	$\theta_w = \frac{V_w}{V_S+V_w+V_a}$ =0.41	—
V_a	Volume of air	$V_a = 2.47$	cm^3
V_w	Volume of water	$V_a = 14.49$	cm^3
V_S	Volume of solid	$V_a = 18.38$	cm^3
D	Hydrodynamic dispersion coefficient	$D = \frac{RT}{F^2}\left[\frac{\frac{1}{n^+}+\frac{1}{n^-}}{\frac{1}{\lambda_+^\circ}+\frac{1}{\lambda_-^\circ}}\right] = 1.45 \times 10^{-5}$	$cm^2 \cdot min^{-1}$
R	The universal gas constant	8.314	$J \cdot (mol \cdot K)^{-1}$
T	Absolute temperature	$T = 273 + ℃ = 273 + 25 = 298K$	K
F	Faraday's constant	96,500	$C \cdot eq^{-1}$
n^+	Cation valence	Lead: 2	—
n^-	Anion valence	Chloride: 1	—

λ_+°	The limiting positive ionic conductance	Lead: 69.5		$cm^2 \cdot S \cdot eq^{-1}$
λ_-°	The limiting negative ionic conductance	Chloride: 76.4		$cm^2 \cdot S \cdot eq^{-1}$
v	Average linear water velocity	$v = \frac{Q}{A} = \frac{5\ mL \cdot min^{-1}}{7.07cm^2} = 0.71$		$cm \cdot min^{-1}$
L	Soil column length	5		cm
x	Distance of the pore water flow travel at specific time	$x = t \cdot v$		cm
t	Leaching time	Measured by a timer		min
V_T	Volume of soil column	$V_T = \pi r^2 L = 35.34\ cm^3$		cm^3
r	Radius of the column	1.5		cm
h	Column height	30		cm
Vc	Pore volume	$Vc = \varphi V_T = (0.41 \times 35.34)\ cm^3 = 14.49\ cm^3$		cm^3
φ	Porosity of the soil column	0.41 or 41%		—
Q	Flow rate	5		$mL \cdot min^{-1}$
MR	Effluent mass recovery rate	CH_4 NBs	0.0597	%
		CO_2 NBs	0.1134	
		H_2 NBs	0.0681	
		DI water	0.0380	
K_d	Deposition rate coefficient	CH_4 NBs	0.07974	min^{-1}
		CO_2 NBs	0.0462	
		H_2 NBs	0.07602	
		DI water	0.09252	

References

1. Razavi, N. R.; Ridal, J. J.; de Wit, W.; Hickey, M. B.; Campbell, L. M.; Hodson, P. V., Ebullition rates and mercury concentrations in St. Lawrence river sediments and a benthic invertebrate. *Environ Toxicol Chem* **2013,** *32*, 857-65.
2. Stoppa, F.; Schiazza, M.; Pellegrini, J.; Ambrosio, F. A.; Rosatelli, G.; D'Orsogna, M. R., Phthalates, heavy metals and PAHs in an overpopulated coastal region: Inferences from Abruzzo, central Italy. *Marine pollution bulletin* **2017,** *125*, 501-512.
3. Church, C.; Spargo, J.; Fishel, S., Strong acid extraction methods for "total phosphorus" in soils: EPA Method 3050B and EPA Method 3051. *Agricultural & Environmental Letters* **2017,** *2*, 160037.
4. EPA, METHOD 3051A
   MICROWAVE ASSISTED ACID DIGESTION OF
   SEDIMENTS, SLUDGES, SOILS, AND OILS. EPA: 1996.
5. Teng-Fei, S.; Xiang, L.; Lei, C.; Tao, X.; Ce-Hui, M.; Yan-Wen, L.; Quan-Ying, C.; Guo-Cheng, H.; De-Chun, H., Research progresses of determination of perfluorinated compounds in environmental water and solid samples. *Chinese Journal of Analytical Chemistry* **2017,** *45*, 601-610.
6. Berry, J. D.; Neeson, M. J.; Dagastine, R. R.; Chan, D. Y.; Tabor, R. F., Measurement of surface and interfacial tension using pendant drop tensiometry. *Journal of colloid and interface science* **2015,** *454*, 226-237.
7. Bashforth, F.; Adams, J. C., *An attempt to test the theories of capillary action by comparing the theoretical and measured forms of drops of fluid*. University Press: 1883.
8. Morita, A.; Carastan, D.; Demarquette, N., Influence of drop volume on surface tension evaluated using the pendant drop method. *Colloid and Polymer Science* **2002,** *280*, 857-864.
9. Wen, C. In *YH Yu. Mechanics of fluidization*, Chemical Engineering Progress Symposium Series, 1966; pp 100-111.
10. New York State Department of Environmental Conservation, Guidelines for conducting bird and bat studies at commercial wind energy projects. Division of Fish Wildlife and Marine Resources, Ed. Albany, NY, 2009.
11. Fernandez, J. F.; Jastorff, B.; Stormann, R.; Stolte, S.; Thoming, J., Thinking in Terms of Structure-Activity-Relationships (T-SAR): A Tool to Better Understand Nanofiltration Membranes. *Membranes (Basel)* **2011,** *1*, 162-83.
12. Kim, H.; Rao, P. S. C.; Annable, M. D., Determination of effective air-water interfacial area in partially saturated porous media using surfactant adsorption. *Water Resources Research* **1997,** *33*, 2705-2711.
13. Brusseau, M. L., Assessing the Potential Contributions of Additional Retention Processes to PFAS Retardation in the Subsurface. *Science of The Total Environment* **2017,** *613-614*, 43.
14. Nakamura, K.; Yasutaka, T.; Kuwatani, T.; Komai, T., Development of a predictive model for lead, cadmium and fluorine soil-water partition coefficients using sparse multiple linear regression analysis. *Chemosphere* **2017,** *186*, 501-509.
15. Ding, G.; Peijnenburg, W. J. G. M., Physicochemical Properties and Aquatic Toxicity of Poly- and Perfluorinated Compounds. *Critical Reviews in Environmental Science and Technology* **2013,** *43*, 598-678.
16. Zhang, M.; Wei, S.; Dong, S.; Wei, W.; Zhang, Y., Effects of sodium dodecyl sulfate and solution chemistry on retention and transport of biogenic nano-hydroxyapatite in saturated porous media. *Colloids and Surfaces A: Physicochemical and Engineering Aspects* **2023,** *661*, 130956.
